# Understanding the genetic and environmental specificity and overlap between well‐being and internalizing symptoms in adolescence

**DOI:** 10.1111/desc.12376

**Published:** 2015-12-27

**Authors:** Claire M.A. Haworth, Kathryn Carter, Thalia C. Eley, Robert Plomin

**Affiliations:** ^1^ MRC Integrative Epidemiology UnitSchool of Experimental Psychology & School of Social and Community MedicineUniversity of BristolUK; ^2^ Social, Genetic and Developmental Psychiatry CentreKing's College LondonUK

## Abstract

Moderate inverse correlations are typically found between well‐being and mental illness. We aimed to investigate the role of genes and environments in explaining the relationships between two aspects of well‐being and two measures of internalizing symptoms. Altogether, 4700 pairs of 16‐year‐old twins contributed data on subjective happiness and life satisfaction, as well as symptoms of depression and emotional problems. Well‐being was moderately correlated with internalizing symptoms (range = −0.45, −0.58). Multivariate twin model‐fitting indicated both genetic and environmental overlap. Life satisfaction and happiness demonstrated different patterns of overlap, with stronger genetic links between life satisfaction and depression. Non‐shared environmental influences were largely specific to each trait. This study supports the theory of mental health and illness being partly (but not entirely) correlated dimensions. There are also significant genetic and environmental factors to identify for well‐being that go beyond the absence of mental illness. It is therefore possible that different interventions are needed for treating mental illness and promoting mental health.

## Research highlights

- Genetic influences explained on average 40% of the −0.51 phenotypic correlation between well‐being and internalizing symptoms.
- Life satisfaction has stronger genetic links with depression than does happiness, despite similar phenotypic correlations between the constructs.
- There are genetic and environmental factors for well‐being that go beyond the absence of symptoms of mental illness: 45% of the genetic influences on life satisfaction and 70% of the genetic influences on happiness are independent of those on internalizing symptoms.

## Introduction

The World Health Organization states that health is ‘not merely the absence of disease’ (WHO, 2006). So how does mental health relate to symptoms of mental illness? Can we use indices of mental illness to tell us about mental health, or use measures of positive mental health to inform us about vulnerability to mental illness? Measures of well‐being typically show moderate negative correlations with measures of mental illness in the range of −0.50 (Keyes, 2002). Nevertheless, it is possible to exhibit both high levels of well‐being and mental illness, or show little sign of mental illness but still score low on well‐being (Greenspoon & Saklofske, 2001). At the extreme, symptoms of positive mental health can be construed as symptoms of mental illness, including positive emotion as part of symptoms of mania in bipolar disorder (Gruber, 2011).

Twin studies allow us to explore what role shared genetic and environmental influences play in the relationship between traits (Plomin, DeFries, Knopik & Neiderhiser, 2013). In the case of twin studies of the relationship between internalizing symptoms and well‐being, no study to date has focused exclusively on adolescence, which is a crucial developmental stage characterized by decreases in well‐being (Goldbeck, Schmitz, Besier, Herschbach & Henrich, 2007) and increases in internalizing symptoms (Rice, Harold & Thapar, 2002). Most teenagers progress through adolescence without developing symptoms of mental illness, yet we have limited understanding of the etiology of positive mental health in adolescence, or of the etiological links with symptoms of mental illness during this period. Understanding positive development more clearly should help us to design intervention and prevention programmes that allow more young people to progress successfully through the considerable biological and social challenges of puberty and adolescence (Goldbeck *et al*., 2007). Evidence from twin studies of internalizing symptoms suggests that genetic influences increase between childhood and adolescence (Rice *et al*., 2002; Eley & Stevenson, 1999). And there is some evidence of neurobiological and treatment efficacy differences between adolescent and adult depression (Kaufman, Martin, King & Charney, 2001), highlighting the need to study these traits within developmentally specific age ranges.

Adult twin studies of the etiological links between internalizing symptoms and well‐being have shown a mixed pattern of results. The highest degree of genetic overlap was found in a sample of 670 pairs of middle‐aged twins (Kendler, Myers, Maes & Keyes, 2011). This study found that 86% of the −0.54 correlation between internalizing disorders and mental well‐being was explained by genetic factors. The phenotypic correlation of −0.54 indicates there were also residual influences; in fact half of the genetic influences on mental well‐being were independent of internalizing disorders.

Genetic influences also explained most (74%) of the phenotypic correlation in a sample of around 1385 twin pairs aged 21–28 years, with a diagnostic measure of life‐time depression, and a dispositional measure of life satisfaction (Nes, Czajkowski, Røysamb, Ørstavik, Tambs *et al*., 2013). However, the phenotypic correlation was lower (−0.36), which may reflect the difference of 6–7 years between assessments in this study. In both of these studies (Kendler *et al*., 2011; Nes *et al*., 2013) a categorical measure of depression was used in contrast to more continuous scales designed to assess symptoms of mental illness in the general population. A previous study by Nes and colleagues (Nes, Czajkowski, Røysamb, Reichborn‐Kjennerud & Tambs, 2008), which did use self‐reported symptom scales of anxiety and depression in a larger sample of 3334 twin pairs, found that genetic factors explained a much lower proportion of the phenotypic correlation (24% in females, 51% in males), even though the samples in the two Nes studies partially overlap. These results suggest that we should expect more modest genetic overlap in the present study where we have used dimensional scores of internalizing symptoms.

There is some indication that different constructs of well‐being may show different patterns of overlap with internalizing symptoms. In the Kendler *et al*. (2011) study, described above, there were some genetic influences that were specific to the emotional well‐being (which includes affect and satisfaction) and social well‐being scales, but not for the psychological well‐being scale. The multidimensional nature of genetic and environmental influences on well‐being, and the overlap with symptoms of mental illness, has also been demonstrated in a study of 613 pairs of middle‐aged male twins (Franz, Panizzon, Eaves, Thompson, Lyons *et al*., 2012), which used four measures that span both hedonic and eudaimonic aspects of well‐being. The authors demonstrated that different constructs of well‐being have varying overlap with depression. In the present study, we will examine whether there are similar differences in genetic overlap for different constructs of well‐being and internalizing symptoms during adolescence.

We are aware of only one twin study that included some adolescents within a sample of a wide age range (12–20 years) (Bartels, Cacioppo, Beijsterveldt & Boomsma, 2013). They report that genetic influences explain 58% of a −0.43 correlation between subjective well‐being and internalizing symptoms in males, and 66% of the −0.58 correlation in females. The subjective well‐being measure, however, was only analysed as an overall composite combining measures of life satisfaction, happiness, and quality of life.

Evidence from phenotypic studies indicates that life satisfaction and affect have different correlates and predictors (Diener, 1994), which seems reasonable given that they represent a cognitive evaluation of one's life versus a more transitory assessment of one's affective states. It is therefore possible that these constructs would show different etiological overlap with internalizing symptoms. The tripartite model of depression and anxiety (Clark & Watson, 1991) suggests that although negative affect is general to both anxiety and depression, a lack of positive affect is specific to depression, so we might predict stronger negative associations between happiness and depression. Our study is the first to investigate the etiological overlap with internalizing symptoms for these cognitive and affective components of well‐being separately. Finding specificity in the overlap between different aspects of well‐being and internalizing symptoms may help us identify targets for interventions, including those that could improve well‐being in those suffering from mental illness, as well as identify possible cognitive and affective discontinuities between what we consider to be healthy behaviour and what we classify as symptoms of illness.

### Present study

We aimed to investigate the role of genes and environments in explaining the relationships between two aspects of well‐being and two measures of internalizing symptoms using a large adolescent sample of twins. Our study is the first to date that focuses specifically on understanding the etiology of these relationships during adolescence.

## Method

### Sample

The Twins Early Development Study (TEDS) is a longitudinal study of twins born in England and Wales between 1994 and 1996 (Haworth, Davis & Plomin, 2013). TEDS is reasonably representative of the general population in terms of parental education, ethnicity and employment status (Haworth *et al*., 2013). Zygosity was assessed through a parent questionnaire of physical similarity (Price, Freeman, Craig, Petrill, Ebersole *et al*., 2000). For cases where zygosity was unclear, DNA testing was conducted.

TEDS families were invited to participate in the 16‐year study, and parents provided informed consent. Approval for TEDS has been provided by the Institute of Psychiatry Research Ethics Committee. The current paper focuses on self‐report measures from a postal questionnaire. The mean age at assessment was 16.32 (*SD *= .68). In all, 9463 individuals provided well‐being data, including 4697 complete twin pairs; Supplementary Table 1 provides details about the composition of the sample.

### Measures

Our questionnaire booklet included the Subjective Happiness Scale (SHS; Lyubomirsky & Lepper, 1999) and the Brief Multidimensional Student Life Satisfaction Scale (Seligson, Huebner & Valois, 2003). The SHS consists of four items (one negatively worded) on a 7‐point scale, e.g. ‘*Some people are generally very happy. They enjoy life regardless of what is going on, getting the most out of everything. To what extent does this describe you?*’ The life satisfaction scale consists of six items assessing satisfaction with family, friends, school, self, where you live, and overall satisfaction with life, all rated on a 7‐point scale. Item scoring was reversed where necessary so that a higher score denoted greater well‐being, and we calculated the composites by taking the mean of the items (requiring 50% of the items to be non‐missing).

Both measures demonstrated good internal consistency reliability, with alphas of .79 for happiness and .86 for life satisfaction. A subsample of 2000 twin pairs completed the same subjective happiness scale, and a variation on an overall life satisfaction scale twice, roughly six months apart. Test–retest correlations for these measures were high: .68 for subjective happiness, and .63 for the global life satisfaction scale.

To assess internalizing symptoms we included the short Mood and Feelings Questionnaire (MFQ; Angold, Costello, Messer, Pickles, Winder *et al*., 1995) to measure symptoms of depression and the emotional subscale of the Strengths and Difficulties questionnaire (Goodman, 2001) as an index of emotional symptoms. The MFQ consists of 13 items on a 3‐point scale. The twins were asked to rate how true statements e.g. ‘*I didn't enjoy anything at all*’ were for them over the past two weeks. This scale demonstrated good internal consistency reliability (0.88). This measure has shown reasonable validity compared to clinical diagnoses of depression (Thapar & McGuffin, 1998). The emotional subscale consists of five items on the same 3‐point scale, asking about experiences in the past six months, e.g. ‘*I worry a lot*’. This scale showed reasonable internal consistency reliability for a short scale (0.69). We calculated the composites by taking the mean of the items (requiring 50% of the items to be non‐missing); higher values denote more internalizing symptoms.

### Data preparation

All of the measures were skewed: subjective happiness had a skew of −.52; life satisfaction −1.12; depression 1.95; and emotional symptoms .75. A van der Waerden rank transformation was applied to all measures. Analyses repeated on untransformed data indicated the same pattern of results. In addition, as is standard in twin analyses, all measures were corrected for the mean effects of age and sex using a regression procedure (McGue & Bouchard, 1984).

### Twin analyses

Twin analyses allow the estimation of the relative contributions of genes and environments to individual differences in measured traits (Plomin *et al*., 2013). Twin intraclass correlations were calculated (Shrout & Fleiss, 1979), providing an initial indication of additive genetic (A), shared environmental (C), and non‐shared environmental (E) factors. Additive genetic influence, also commonly known as heritability, is estimated as twice the difference between the identical and fraternal twin correlations. The contribution of the shared environment, which makes members of a family similar, is estimated as the difference between the identical twin correlation and heritability. Non‐shared environments (environments specific to individuals) are estimated by the difference between the identical twin correlation and 1 because they are the only source of variance making identical twins different. Estimates of the non‐shared environment also include measurement error.

Structural equation model‐fitting allows more complex analyses, formal tests of significance and the calculation of confidence intervals (Rijsdijk & Sham, 2002). A Cholesky decomposition was fitted to the data using Mx (Neale, Boker, Xie & Maes, 2006). Here we focus on the Cholesky results, but alternative multivariate models were tested; these are described in the Supplementary Information. The Cholesky decomposition allows the investigation of overlap and specificity in the genetic and environmental influences on our measures. The first genetic factor (A1) represents genetic influences on depression. The extent to which these same genes also influence emotional symptoms, life satisfaction and happiness is also estimated, and is represented by the diagonal pathways from A1 to the other variables. The second genetic factor (A2) represents genetic influences on emotional symptoms that are independent of those influencing depression. The extent to which these genes also influence life satisfaction and happiness is also estimated. The third genetic factor (A3) indexes genetic influences on life satisfaction that are independent of genetic influences shared with depression and emotional symptoms. The impact of these genes on happiness is also estimated. Finally, the fourth genetic factor (A4) represents residual genetic influences on happiness. The same decomposition is done for the shared environmental and non‐shared environmental influences (C1–4 and E1–4, respectively). The order in which the variables are included in the analyses is arbitrary; we chose to include the internalizing measures first, followed by well‐being because this investigation is focused on whether there are genetic and environmental influences on well‐being over and above those that influence internalizing symptoms. However, we also converted the results to the mathematically equivalent correlated factors solution, which does not impose an order on the variables; rather, it focuses on each bivariate combination of variables, providing estimates of the degree of genetic and environmental correlation between each of our measures (see Supplementary Table 4). Finally, it is possible to calculate the proportion of the phenotypic correlation that is accounted for by shared genetic and environmental influences. This statistic is known as bivariate heritability.

## Results

### Descriptive statistics

**Figure 1:**
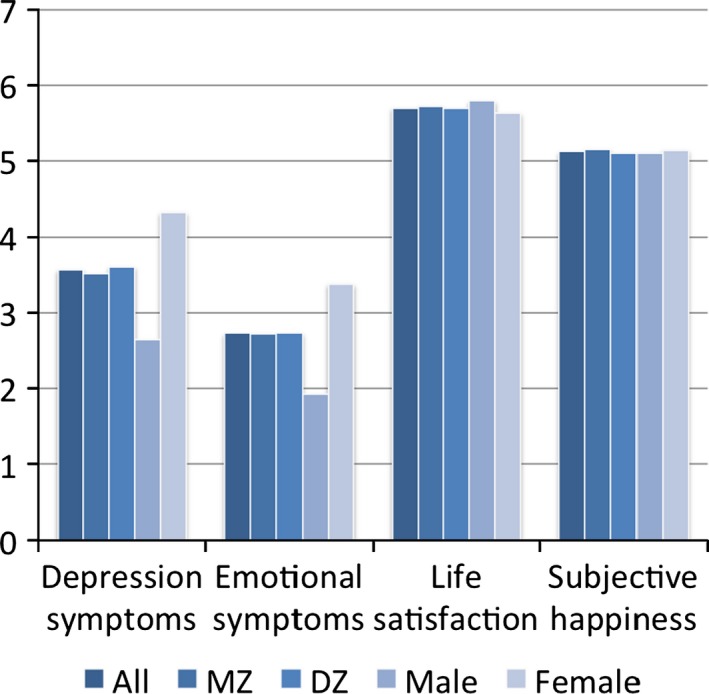
Means split by sex and zygosity. MZ = monozygotic twins; DZ = dizygotic twins. Descriptives are presented for one randomly selected member of each twin pair. *N* values are as follows: All *N* = 4732–4739; MZ *N* = 1694–1698; DZ *N* = 3037–3041; Male *N* = 2116–2120; Female *N* = 2615–2619. Supplementary Table 1 includes further information about the standard deviations and ANOVA to test for differences in sex and zygosity. In the model‐fitting analyses we correct for mean effects of sex using a regression procedure.

There were mean sex differences in life satisfaction, depression and emotional symptoms, with males scoring higher for life satisfaction, and females scoring higher for symptoms of depression and emotional symptoms (Figure 1 and Supplementary Table 1). These effects explained less than 1%, 4%, and 10% of the variance, respectively. There were no sex differences in subjective happiness, and negligible differences between identical and fraternal twins. The correlations between the scales were significant at *p *<* *.001, and in the expected direction (Table 1).

**Table 1:** Phenotypic and twin correlations

	Depression symptoms	Emotional symptoms	Life satisfaction	Subjective happiness
Emotional symptoms	.64			
Life satisfaction	−.58	−.49		
Subjective happiness	−.50	−.45	.61	
MZ (*N*_pairs_ = 1681–1688)	.42	.42	.57	.41
DZ (*N*_pairs_ = 3011–3017)	.27	.17	.33	.21

Note:

All significant at *p *<* *.001; Phenotypic correlations conducted on one randomly selected member of each pair (*N* range = 4725–4739). *N*_pairs_ = number of complete twin pairs. Correlations performed on transformed, age‐ and sex‐corrected measures. Twin correlations split by sex and zygosity are included in Supplementary Table 2, with overlapping confidence intervals for our estimates for same‐ and opposite‐sex fraternal twins, and for our male and female pairs. Therefore we conducted our multivariate analyses on a sample with male and female twins combined.

### Genetically informative analyses

**Figure 2:**
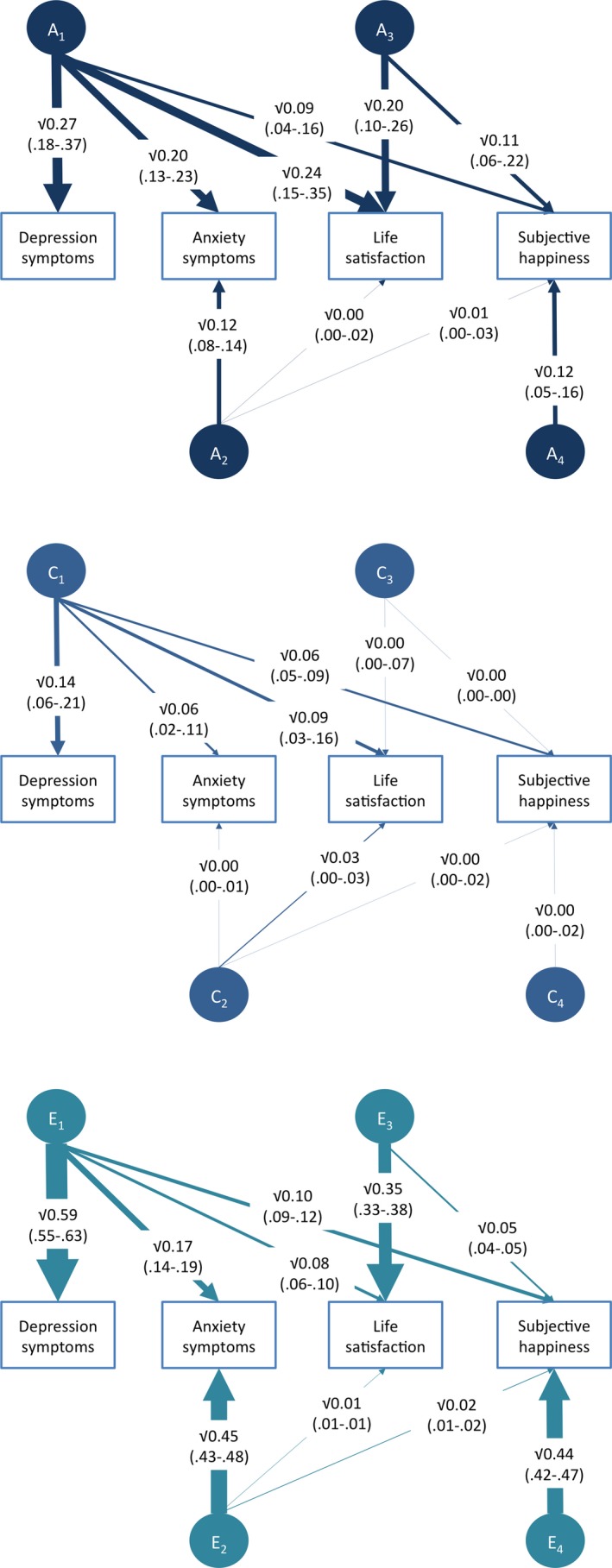
Genetic and environmental estimates from the Cholesky decomposition. A = additive genetic; C = shared environment; E = non‐shared environment. Line weights represent the magnitude of the effect. Dotted lines indicate estimates that have confidence intervals overlapping with zero. 95% confidence intervals are shown in parentheses. *Note*: The Cholesky decomposition provided the best fit. Fit statistics for alternative models are shown in Supplementary Table 5.

In all cases identical twin correlations are greater than the fraternal twin correlations, indicating genetic influence (Table 1). The genetic and environmental influences on each trait, and their confidence intervals, were estimated using structural equation twin models. The estimates of the standardized variance components from the Cholesky decomposition are shown in Figure 2 and Supplementary Table 3.

Heritability ranged from 0.27 to 0.44, with most of the remaining variance explained by non‐shared environmental influences (0.44 to 0.62). Results indicate a mixed pattern of overlap and specificity. For the genetic influences there was a strong first factor for depression that also influenced emotional symptoms and life satisfaction, and to a lesser extent, subjective happiness. There were some genetic influences on emotional symptoms that were independent of those on depression (the A2 factor), and there were significant genetic influences on the well‐being measures that were independent of the genetic influences on the internalizing measures (the A3 and A4 factors). The shared environmental influences were modest, but largely the same influences were important for all four measures, as seen by the C1 factor followed by near‐zero influences for the C2–4 factors. In contrast, most of the non‐shared environmental influences were specific to each measure. There was some overlap in the non‐shared environmental influences as well, and this was captured primarily by the first E1 factor.

**Figure 3:**
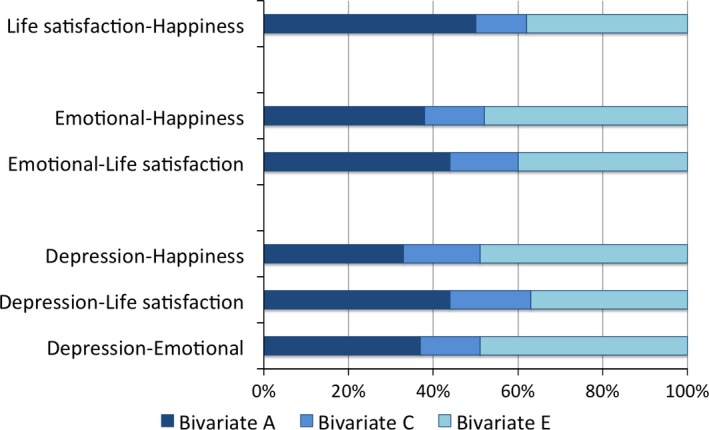
Proportion of the phenotypic correlation explained by overlapping genetic, shared environmental and non‐shared environmental influences. These results focus on what is common between the different measures. The phenotypic correlations (shown in Table 1) are broken down into the proportion explained by overlapping genetic and environmental influences. Bivariate A = Proportion of the phenotypic correlation explained by common genetic influences. Bivariate C = Proportion of the phenotypic correlation explained by common shared environmental influences. Bivariate E = Proportion of the phenotypic correlation explained by common non‐shared environmental influences. Confidence intervals for these estimates are shown in Supplementary Table 4.

Estimates for the proportion of the phenotypic correlations explained by overlapping genetic, shared and non‐shared environmental influences are provided in Figure 3. For the relationship between internalizing measures and life satisfaction, genetic influences explain 44% of the phenotypic correlation, and for internalizing measures and subjective happiness genetic influences explain 33% of the overlap with depression and 38% of the overlap with emotional symptoms.

## Discussion

Our results indicate moderate genetic and environmental overlap between internalizing symptoms and two indices of well‐being in adolescence. As expected, well‐being was moderately correlated with internalizing symptoms, and genetic influences explained between 33 and 44% of the phenotypic correlation. These results support the theory of mental health and illness being partly (but not entirely) correlated dimensions.

Previous studies of this overlap in adults produced mixed findings with 22–86% of the phenotypic correlations being explained by common genetic influences (a statistic called bivariate heritability). Our bivariate heritability ranged from 33–44%, and is therefore towards the lower end of previous results, and most similar to the study by Franz and colleagues (2012), which found bivariate heritability of 37% and a phenotypic correlation of −0.49 between depression and life satisfaction using the same Cholesky decomposition as in our analyses. The differences in the magnitude of bivariate heritability do not appear to be related to the age of the samples (e.g. 37% for twins aged 51–60 years old in Franz *et al*. (2012), and 86% for middle‐aged twins in Kendler *et al*. (2011)), but may be related to the measure of mental illness. The highest estimates for bivariate heritability (86% and 74%) came from the two studies that used categorical measures indicating presence or absence of mental illness (Kendler *et al*., 2011; Nes *et al*., 2013). Further investigation of the differences in overlap between well‐being and diagnostic versus dimensional measures of internalizing symptoms is warranted, especially as one might have predicted stronger links with a dimensional measure that has been designed to capture the range of positive and negative aspects of the trait.

In our study, life satisfaction and happiness demonstrated slightly different patterns of overlap with symptoms of depression and emotional symptoms. This difference is most strikingly seen in the estimates from the Cholesky decomposition (Figure 2). The (squared) influence of the first genetic factor (A1) is 0.24 on life satisfaction, but only 0.09 on happiness. No such pattern is seen for the shared or non‐shared environmental influences. This is the first analysis to show that life satisfaction has stronger genetic links with depression than happiness does. This finding is bolstered by the fact that life satisfaction and subjective happiness are also to some extent etiologically distinct, with genetic and non‐shared environmental influences on happiness that are not shared with life satisfaction. These findings add weight to the argument that future analyses should explore the specificity of overlap, perhaps even at the level of items, to inform us about the nuances of the overlap between mental health and illness, and between measures of well‐being. The tripartite model of depression and anxiety predicts stronger overlap between positive affect and depression compared to anxiety (Clark & Watson, 1991). If we assume that positive affect is more related to happiness (than life satisfaction), then we do not see this pattern in our data, but we do not have a formal measure of positive affect to test this directly, and it is possible that life satisfaction would also be correlated with positive affect. Our finding of a stronger genetic link between life satisfaction and depression does not appear to be driven by similarities in the specific items used. An intriguing future direction would be to investigate whether life satisfaction, which represents a cognitive appraisal of one's life, is specifically linked to cognitive symptoms of depression, and whether changes in life satisfaction could be used as an early marker of these cognitive biases.

### Genetic and environmental specificity

Our results show that mental health is more than the absence of mental illness. Although we find overlap between mental health and illness, there is still variance specific to mental health. These phenotypic findings alone provide support for the study and investigation of mental health as an additional source of information about the human mental condition. Low levels of depression should not be interpreted as good well‐being and, likewise, low well‐being does not mean that an individual is depressed. We already know that positive mental health provides additional benefits to cardiovascular and other indices of physical health over and above the effects of being free from symptoms of mental illness (Steptoe, Wardle & Marmot, 2005). Given the importance of the variance in well‐being that is independent of mental illness, we know surprisingly little about its genetic and environmental origins. Here we show that 45% of the genetic influences on life satisfaction (calculated as the A3 path on life satisfaction divided by the total genetic influence on life satisfaction: .20/(.24 + .00 + .20)) are independent of those on internalizing symptoms. For happiness, the degree of genetic specificity is even higher, with 70% of the genetic influences on happiness being independent of those on internalizing symptoms (calculated as the sum of paths A3 and A4 onto happiness, divided by the total genetic influence on happiness: (.11 + .12)/(.09 + .01 + .11 + .12) = .23/.33). There remains significant genetic influence on happiness even when the shared genetic variance with internalizing symptoms and life satisfaction has been removed (36% calculated by the A4 path onto happiness divided by the total genetic influence on happiness .12/(.09 + .01 + .11 + .12) = .12/.33). Shared environmental influences do not contribute to specificity, but non‐shared environmental influences are important in making internalizing symptoms different from well‐being. As is frequently found, non‐shared environmental influences are largely specific to each and every measure, suggesting that different environmental experiences are relevant for mental health and illness, but also that the environments that create individual differences in happiness are not the same as those that influence life satisfaction.

A potential implication of finding specificity in the influences on mental health and illness is that it is possible that different interventions will be needed for treating mental illness and promoting mental health; our results indicate that these interventions will need to target different biological pathways. Perhaps what is needed is a two‐step intervention that first reduces the symptoms of mental illness, followed by a second stage that focuses on promoting mental health. It has already been shown that the relapse rate for depression is lower in patients who received some form of well‐being therapy for residual symptoms (Fava, Rafanelli, Cazzaro, Conti & Grandi, 1998), with benefits still present at a six‐year follow‐up (Fava, Ruini, Rafanelli, Finos, Conti *et al*., 2004). In addition, positive outcomes, such as life satisfaction, are often the main criterion for assessing recovery from a patient perspective (Frisch, Cornell, Villanueva & Retzlaff, 1992) even though the treatment most patients receive does not specifically target positive mental health. Our findings also imply that lifestyle or public health interventions for promoting positive mental health should not be used as a replacement for more targeted clinical treatment of depression and anxiety. Whether such public health interventions could be used as a preventative measure against the future development of mental illness requires further investigation.

### Limitations

Although there is evidence for the clinical validity of these brief questionnaire measures (e.g. Thapar & McGuffin, 1998), it is possible that measuring well‐being and internalizing symptoms from the same rater contemporaneously may bias our results. Some of the correlation between these measures may be due to within‐rater bias; although this does not explain the differences in overlap between our measures of well‐being and internalizing symptoms. Measurement error is typically subsumed within the non‐shared environmental variance, but if responses to these items are related to a general responding bias then it is possible that this could explain some of the genetic overlap seen in these analyses, but not the differences in genetic overlap. In addition, this study is subject to the usual limitations of the twin design, explained in detail elsewhere (Rijsdijk & Sham, 2002).

## Supporting information


**Table S1.** Means (SD) and ANOVA results.
**Table S2.** Twin intraclass correlations split by sex and zygosity (95% confidence interval).
**Table S3.** Estimates of standardized variance components (95% confidence intervals) of genetic and environmental influence from the Cholesky decomposition.
**Table S4.** Genetic and environmental correlations and bivariate heritability, shared and non‐shared environmental overlap.
**Table S5.** Fit statistics for multivariate twin models.
**Data S1.** Description of alternative multivariate twin models.
