# Supplementary material for: Understanding the genetic and environmental specificity and overlap between well‐being and internalizing symptoms in adolescence
Source: Dev Sci. 2015 Dec 27;20(2):e12376. doi: 10.1111/desc.12376 (PMC5347864; doi:10.1111/desc.12376)
Supplement: Supplementary file 1 — Table S1. Means (SD) and ANOVA results. Table S2. Twin intraclass correlations split by sex and zygosity (95% confidence interval). Table S3. Estimates of standardized variance components (95% confidence intervals) of genetic and environmental influence from the Cholesky decomposition. Table S4. Genetic and environmental correlations and bivariate heritability, shared and non‐shared environmental overlap. Table S5. Fit statistics for multivariate twin models. Data S1. Description of alternative multivariate twin models. [file DESC-20-na-s001.docx]

**Supplementary Information for:**

**Understanding the genetic and environmental specificity and overlap between wellbeing and internalising symptoms in adolescence**

Contents:

Page 2: Supplementary Table 1: Means (SD) and ANOVA results

Page 3: Supplementary Table 2: Twin intraclass correlations split by sex and zygosity (95% confidence interval)

Page 4: Supplementary Table 3: Estimates of standardized variance components (95% confidence intervals) of genetic and environmental influence from the Cholesky decomposition

Page 6: Supplementary Table 4: Genetic and environmental correlations and bivariate heritability, shared and non-shared environmental overlap

Page 8: Description of alternative multivariate twin models

Page 10: Supplementary Table 5: Fit statistics for multivariate twin models

**Supplementary Table 1: Means (SD) and ANOVA results**

|  | Depression symptoms | Emotional symptoms | Life satisfaction | Subjective happiness |
| --- | --- | --- | --- | --- |
| All  (N=4732-4739) | 3.57  (4.38) | 2.73  (2.23) | 5.70  (1.06) | 5.12  (.96) |
| MZ  (N=1694-1698) | 3.52  (4.42) | 2.72  (2.27) | 5.72  (1.08) | 5.15  (.96) |
| DZ  (N=3037-3041) | 3.61  (4.36) | 2.73  (2.20) | 5.69  (1.05) | 5.10  (.96) |
| Male  (N=2116-2120) | 2.64  (3.50) | 1.92  (1.83) | 5.79  (1.01) | 5.10  (.94) |
| Female  (N=2615-2619) | 4.33  (4.86) | 3.38  (2.30) | 5.63  (1.09) | 5.14  (.98) |
| Sex p-value | <.001 | <.001 | <.001 | .356 |
| Sex effect size | 0.040 | 0.104 | 0.007 | <0.001 |
| Zygosity p-value | .045 | .091 | .045 | .075 |
| Zygosity effect size | 0.001 | 0.001 | 0.001 | 0.001 |

N=one randomly selected member of each pair; ANOVA conducted on transformed scales; effect size=eta squared.

**Supplementary Table 2: Twin intraclass correlations split by sex and zygosity (95% confidence interval)**

|  | Depression symptoms | Emotional symptoms | Life satisfaction | Subjective happiness |
| --- | --- | --- | --- | --- |
| MZ | 0.42  (.38-.46)  N = 1688 | 0.42  (.38-.46)  N = 1684 | 0.57  (.53-.60)  N = 1682 | 0.41  (.37-.45)  N = 1681 |
| DZss | 0.31  (.27-.36)  N = 1536 | 0.18  (.13-.23)  N = 1531 | 0.37  (.33-.41)  N = 1532 | 0.25  (.20-.30)  N = 1533 |
| DZos | 0.23  (.18-.27)  N = 1481 | 0.17  (.12-.21)  N = 1480 | 0.29  (.24-.33)  N = 1480 | 0.16  (.11-.21)  N = 1483 |
| DZall | 0.27  (.24-.30)  N = 3017 | 0.17  (.14-.21)  N = 3011 | 0.33  (.30-.36)  N = 3012 | 0.21  (.17-.24)  N = 3016 |
| MZM | 0.37  (.30-.43)  N = 696 | 0.38  (.31-.44)  N = 692 | 0.52  (.46-.57)  N = 693 | 0.43  (.37-.49)  N = 691 |
| MZF | 0.44  (.39-.49)  N = 992 | 0.44  (.39-.49)  N = 992 | 0.60  (.55-.63)  N = 989 | 0.41  (.35-.46)  N = 990 |
| DZM | 0.23  (.16-.30)  N = 655 | 0.12  (.04-.19)  N = 654 | 0.35  (.28-.42)  N = 653 | 0.19  (.11-.26)  N = 656 |
| DZF | 0.36  (.30-.42)  N = 881 | 0.23  (.16-.29)  N = 877 | 0.39  (.33-.44)  N = 879 | 0.30  (.24-.36)  N = 877 |

N = complete twin pairs; MZ = monozygotic twins; DZss = dizygotic same-sex twins; DZos = dizygotic opposite sex twins; DZall = dizygotic twins (same-sex and opposite-sex combined); MZM = monozygotic male twins; MZF = monozygotic female twins; DZM = dizygotic male twins; DZF = dizygotic female twins.

**Supplementary Table 3: Estimates of standardized variance components (95% confidence intervals) of genetic and environmental influence from the Cholesky decomposition**

|  | Depression symptoms | Emotional symptoms | Life satisfaction | Subjective happiness |
| --- | --- | --- | --- | --- |
| A1 | 0.27  (.18-.37) | 0.20  (.13-.23) | 0.24  (.15-.35) | 0.09  (.04-.16) |
| A2 |  | 0.12  (.08-.14) | 0.00  (.00-.02) | 0.01  (.00-.03) |
| A3 |  |  | 0.20  (.10-.26) | 0.11  (.06-.22) |
| A4 |  |  |  | 0.12  (.05-.16) |
| A | 0.27  (.18-.37) | 0.32  (.25-.38) | 0.44  (.36-.52) | 0.34  (.26-.40) |
|  |  |  |  |  |
| C1 | 0.14  (.06-.21) | 0.06  (.02-.11) | 0.09  (.03-.16) | 0.06  (.05-.09) |
| C2 |  | 0.00  (.00-.01) | 0.03  (.00-.03) | 0.00  (.00-.02) |
| C3 |  |  | 0.00  (.00-.07) | 0.00  (.00-.00) |
| C4 |  |  |  | 0.00  (.00-.02) |
| C | 0.14  (.06-.21) | 0.06  (.02-.11) | 0.11  (.05-.18) | 0.06  (.02-.12) |
|  |  |  |  |  |
| E1 | 0.59  (.55-.63) | 0.17  (.14-.19) | 0.08  (.06-.10) | 0.10  (.09-.12) |
| E2 |  | 0.45  (.43-.48) | 0.01  (.01-.01) | 0.02  (.01-.02) |
| E3 |  |  | 0.35  (.33-.38) | 0.05  (.04-.05) |
| E4 |  |  |  | 0.44  (.42-.47) |
| E | 0.59  (.55-.63) | 0.62  (.59-.66) | 0.45  (.42-.48) | 0.60  (.57-.64) |

A/C/E=total additive genetic/shared environmental/non-shared environmental influence. A1/C1/E1=genetic/shared environmental/non-shared environmental influence on first trait and its influence on the remaining traits. A2/C2/E2= influence on second trait (independent of influences shared with first trait) and its influence on the remaining traits. A3/C3/E3= influence on third trait (independent of influences shared with first and second trait) and its influence on the remaining trait. A4/C4/E4= influence specific to last trait. The total estimate for A/C/E may differ slightly from the sum of the A/C/E1-4 due to rounding.

**Supplementary Table 4: Genetic and environmental correlations and bivariate heritability, shared and non-shared environmental overlap**

|  |  | **rA** | **rC** | **rE** |
| --- | --- | --- | --- | --- |
| Depression symptoms | Emotional symptoms | 0.80  (.71,.88) | 0.99  (.88,1.00) | 0.52  (.49,.55) |
|  | Life satisfaction | -0.73  (-.86,-.61) | -0.87  (-1.00,-.63) | -0.42  (-.46,-.39) |
|  | Subjective happiness | -0.53  (-.65,-.38) | -0.99  (-1.00,-.80) | -0.40  (-.43,-.36) |
| Emotional symptoms | Life satisfaction | -0.56  (-.65,-.46) | -0.93  (-1.00,-.65) | -0.36  (-.40,-.32) |
|  | Subjective happiness | -0.51  (-.60,-.43) | -1.00  (-1.00,-.73) | -0.34  (-.38,-.31) |
| Life satisfaction | Subjective happiness | 0.77  (.70,.86) | 0.91  (.58,1.00) | 0.44  (.41,.48) |
|  |  |  |  |  |
|  |  | **Bivariate h2** | **Bivariate c2** | **Bivariate e2** |
| Depression symptoms | Emotional symptoms | 0.37  (.26,.47) | 0.14  (.06,.22) | 0.49  (.45,.54) |
|  | Life satisfaction | 0.44  (.32,.55) | 0.19  (.09,.28) | 0.37  (.33,.42) |
|  | Subjective happiness | 0.33  (.19,.47) | 0.18  (.08,.29) | 0.49  (.43,.54) |
| Emotional symptoms | Life satisfaction | 0.44  (.32,.56) | 0.16  (.07,.26) | 0.40  (.35,.45) |
|  | Subjective happiness | 0.38  (.26,.50) | 0.14  (.06,.23) | 0.48  (.42,.54) |
| Life satisfaction | Subjective happiness | 0.50  (.39,.59) | 0.12  (.05,.20) | 0.38  (.34,.42) |

rA/rC/rE=genetic/shared environmental/non-shared environmental correlation. Bivariate h2 (heritability), c2 (shared environment) and e2 (non-shared environment): proportion of the phenotypic correlation that is explained by genetic, shared and non-shared environmental links.

The results were converted to the correlated factors solution to provide estimates of genetic and environmental correlations between the different measures. These results again highlight the differences in the overlap with depression for the two aspects of wellbeing: life satisfaction shares more genes with depression than subjective happiness does, despite similar phenotypic correlations between the constructs. In contrast, the genetic overlap between the two wellbeing measures and emotional symptoms is roughly equal. Shared environmental correlations were high for all of the measures, and the non-shared environmental correlations were moderate.

**Description of alternative multivariate twin models**

A series of standard multivariate genetic models were fitted to the data using Mx (Neale, Boker, Xie, & Maes, 2006), to assess which provided the best fit. Model fit was compared using Akaike’s Information Criterion, and a likelihood ratio test. The Cholesky decomposition was our base model, as it decomposes as many latent A, C and E factors as there are variables. The Cholesky decomposition is described in detail in the main text. Two further multivariate twin models were fitted to the data: the independent pathway model, and the common pathway model. These models are reduced versions of the Cholesky decomposition, so we can test the difference in fit between these nested models and the Cholesky.

*Independent Pathway Model*

The independent pathway model is more stringent than the Cholesky decomposition. It includes a single set of genetic and environmental factors that influence every variable to a lesser or greater extent, denoted as A_c_, C_c_ and E_c_ (C for common) in the figure below. In addition, there are residual (specific) effects on each variable (A_s_, C_s_, and E_s_).

**Supplementary Figure 1: Independent Pathway Model**


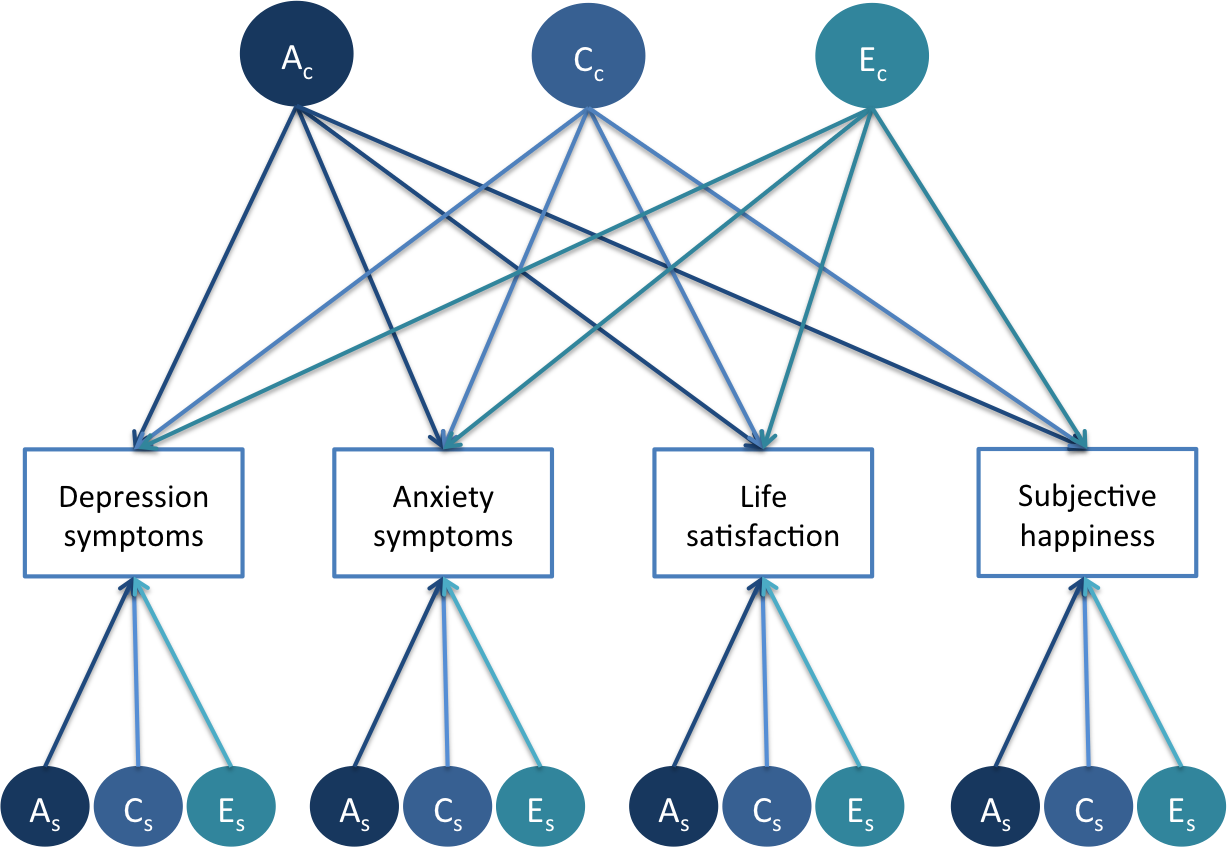


*Common Pathway Model*

The common pathway model tests whether the common genetic and environmental influences work in a unified way through a latent composite of the variables. The common pathway model also allows residual genetic and environmental influences on each variable. In the figure below the common influences are denoted by A_c_, C_c_ and E_c,_ and the specific influences on each trait as A_s_, C_s_, and E_s_.

**Supplementary Figure 2: Common Pathway Model**


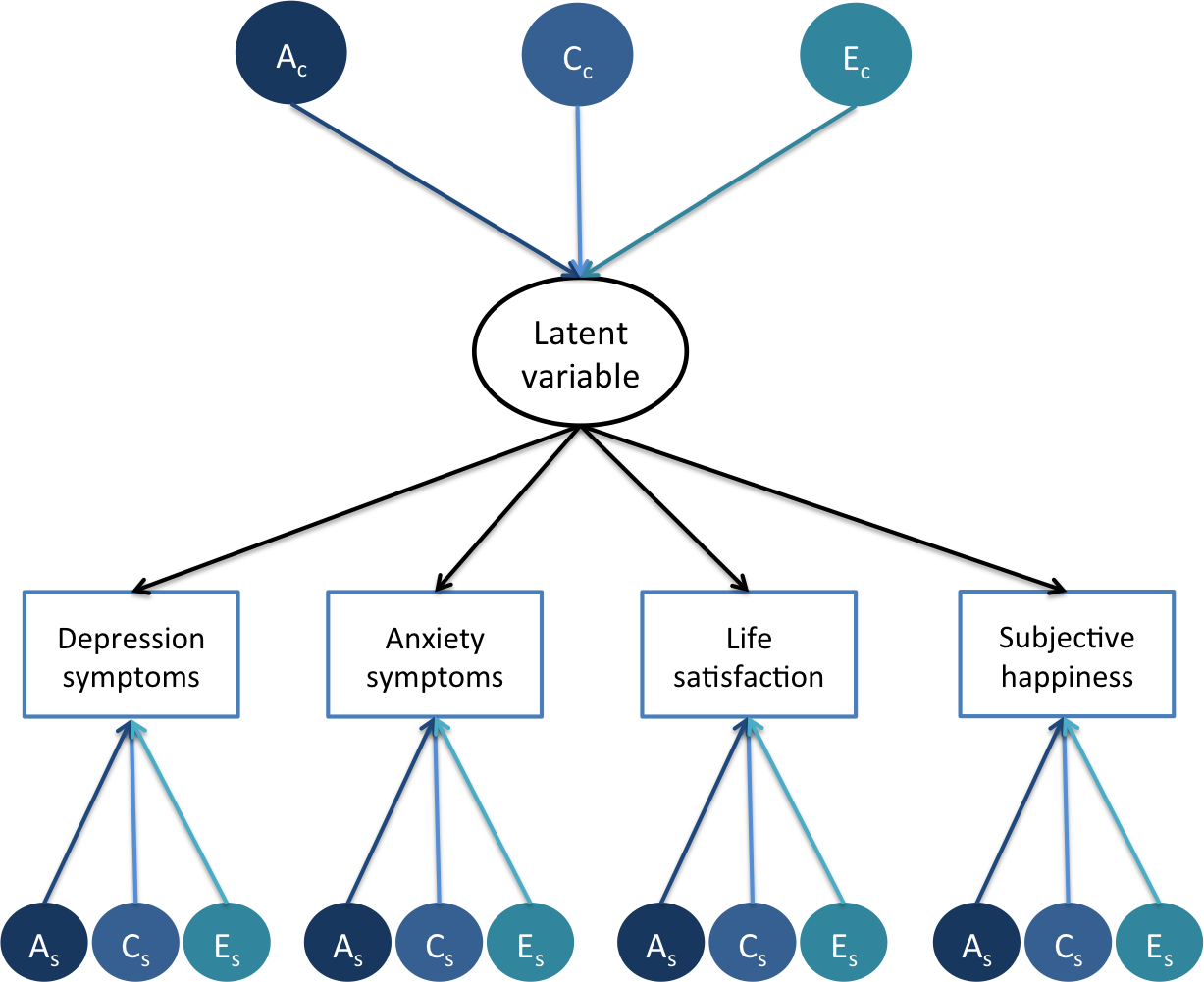


**Supplementary Table 5: Fit statistics for multivariate twin models**

|  | -2ll | df | Δ AIC  compared to saturated | p-value compared to saturated | Δ-2ll  compared to Cholesky | Δ df  compared to Cholesky | Δ AIC  compared to Cholesky | p-value compared to Cholesky |
| --- | --- | --- | --- | --- | --- | --- | --- | --- |
| Saturated model | 91736.092 | 37767 | --- | --- | --- | --- | --- | --- |
| Cholesky decomposition | 91804.305 | 37809 | -15.787 | 0.006 | --- | --- | --- | --- |
| Independent pathway | 91956.163 | 37815 | 124.071 | <0.001 | 151.858 | 6 | 139.858 | <0.001 |
| Common pathway | 92752.243 | 37821 | 908.151 | <0.001 | 947.938 | 12 | 923.938 | <0.001 |

Note. -2ll = minus twice the log likelihood; df = degrees of freedom; AIC = Akaike’s Information Criterion.

The best-fitting genetic model was the Cholesky decomposition. This was selected based on AIC, with lower values indicating better fit. P-values relate to a likelihood ratio test, which is extremely sensitive with sample sizes over about 300 individuals.
